# Acrylic Resin Filling Cell Lumen Enabled Laminated Poplar Veneer Lumber as Structural Building Material

**DOI:** 10.3390/polym14235277

**Published:** 2022-12-02

**Authors:** Xudong Gao, Yiliang Liu, Yanran Qi, Ruizhi Gong, Fengbiao Yao, Jiajia Luo, Yueying Zhao, Yong Dai, Jinguo Wang, Chenglong Lian, Xiaoying Dong, Yongfeng Li

**Affiliations:** 1 Key Laboratory of State Forestry Administration for Silviculture of the Lower Yellow River, College of Forestry, Shandong Agricultural University, Tai’an 271018, China; 2 Postdoctoral Innovation Practice Base, Shandong Xiaguang Group Co., Ltd., Jining 277600, China; 3 Jiangsu Longyuan Decoration Materials Co., Ltd., Suqian 223900, China; 4 Heze Forestry Administration of Shandong Province, Heze 274099, China

**Keywords:** poplar wood, laminated veneer lumber, acrylic, curing agent, filling, cell cavity, mechanical properties, dimensional stability

## Abstract

Wood is a viable alternative to traditional steel, cement, and concrete as a structural material for building applications, utilizing renewable resources and addressing the challenges of high energy consumption and environmental pollution in the construction industry. However, the vast supply of fast-growing poplar wood has bottlenecks in terms of low strength and dimensional stability, making it difficult to use as a structural material. An environmentally friendly acrylic resin system was designed and cured in this study to fill the poplar cell cavities, resulting in a new type of poplar laminated veneer lumber with improved mechanical strength and dimensional stability. The optimized acrylic resin system had a solid content of 25% and a curing agent content of 10% of the resin solid content. The cured filled poplar veneer gained 81.36% of its weight and had a density of 0.69 g/cm^3^. The static flexural strength and modulus of elasticity of the further prepared laminated veneer lumber were 123.12 MPa and 12,944.76 MPa, respectively, exceeding the highest flexural strength required for wood structural timber for construction (modulus of elasticity 12,500 MPa and static flexural strength 35 MPa). Its tensile strength, impact toughness, hardness, attrition value, water absorption, water absorption thickness expansion, and water absorption width expansion were 58.81%, 19.50%, 419.18%, 76.83%, 44.38%, 13.90%, and 37.60% higher than untreated laminated veneer lumber, demonstrating improved mechanical strength and dimensional stability, significantly. This method provides a novel approach to encouraging the use of low-value-added poplar wood in high-value-added structural building material applications.

## 1. Introduction

Today’s world, supported by non-renewable resources, is facing severe challenges such as energy, environment and climate change. It has become a broad social consensus to develop and use renewable resources efficiently to replace non-renewable resources and solve the crisis. In the construction sector, non-renewable resources such as steel, cement and concrete are used as the primary material, emitting considerable amounts of CO_2_ during the processing, affecting the environmental climate and consuming large amounts of energy. According to statistics, energy consumption in the building sector reaches around 40% of the total global energy consumption [1,2,3,4,5]. Therefore, the extensive use of green and renewable resources in the construction sector is a decisive step toward promoting sustainable social development. As one of the largest biomass resources, wood is recognized as a green and low-carbon material with the advantages of recycling, high specific strength, thermal insulation and beautiful textures [6,7,8,9,10]. If it is widely used in the construction field, it will definitely promote energy saving and utilization and environmental climate improvement. However, with the severe shortage of high-quality timber resources in the world, although the fast-growing forest timber (e.g., poplar and pine) as a substitute is rich in resources, it has the bottleneck problem of low strength [11,12] and unstable size [13,14], which restricts its extensive and efficient use in the field of building structures. Although some lightweight structural timbers such as oriented strand board (OSB) [15,16,17,18], laminated veneer lumber (LVL) [19,20,21,22] and cross-laminated timber (CLT) [23,24,25,26] have been developed through structural unit reorganization and can be used as building materials, these raw materials are mostly limited to pine resources, and poplar resources with relatively lower strength and dimensional stability are not yet available for direct use as structural building materials through unit reorganization.

In general, filling the cell cavity with resin can improve both the strength and dimensional stability of the wood [27,28]. However, commonly used resins are aldehydes, such as phenolic (PF) [29,30,31,32], urea-formaldehyde (UF) [33,34,35] and melamine (MF) [36,37,38,39] resins. The slow release of formaldehyde, which in turn endangers the environment and human health, restricts the popularization and application of this type of technology. Therefore, the development of an environmentally friendly resin system for impregnating and filling the cell lumen is a breakthrough to solve the bottleneck problem of poplar. Water-based acrylic resins [40,41,42,43,44,45,46,47], furfuryl alcohol resins [48,49,50,51], polyester resins [52,53,54] and 2D resins [55,56,57] have been studied as environmentally friendly filler modifiers to replace aldehyde resins [58].

Most of all, acrylic resins exhibit good bending resistance [40,41,42,43,44,45], compressive properties [41,42,43], surface hardness [40,41,43] and dimensional stability [42,43], due to their relatively high molecular weight. However, two difficulties remain in these resins: (1) the resin needs to be fitted with a suitable curing agent to fill the cell cavity firmly, and matching the correct curing agent is a difficult task; (2) although the high molecular weight of the resin is beneficial to wood modification, it tends to lead to the high viscosity of the resin liquid, especially for large-size solid wood, which is difficult to penetrate and fill evenly. Therefore, designing an acrylic resin with the proper viscosity to effectively penetrate and fill the wood is another difficulty in achieving this type of resin reinforcement and dimensional stabilization of wood.

In this study, a resin system with suitable viscosity for curing and filling in the cell cavity was constructed by regulating the solid content of the resin and the amount of curing agent and matching the alkaline environment. The laminated composite material was based on modified poplar veneer as a structural unit, impregnating it with resin and then laminating it in the same direction as each other to reconstitute it for use as a structural material in buildings. Thus, breaking the bottleneck faced by acrylic resin in effectively filling large-sized wood. The resin-filled laminated timber had a resin weight percent gain (WPG) of 81.36% and a density of 0.705 g/cm^3^ at a thickness of 1.5 cm, which was 80.31% higher than the untreated sample. The static bending strength and elastic modulus reached 123.13 MPa and 12,944.76 MPa, respectively, which reached the highest grade of structural timber for buildings. The thickness swelling rate and width swelling rate was 2.95% and 3.51%, an improvement of 13.90% and 37.60% compared to untreated material. This resin system formulation design and modification unit reorganization strategy provide new ideas for the use of poplar wood in high-value-added structural building materials areas.

## 2. Materials and Methods

### 2.1. Materials

Poplar rotary cut veneer (thickness 3 mm, average density 0.35 g/cm^3^) was purchased from Tai’an Wood Market (Tai’an, China). Acrylic resin particle (molecular formula C_3_H_4_O_2_, molecular weight 1700, model Joncryl 682) was obtained from Germany BASF SE Group (Shanghai, China). Triethylamine (molecular formula N(C_2_H_5_)_3_, analytically pure) was purchased from Tianjin Kaitong Chemical Reagent Co., Ltd. (Tianjin, China). Propidium curing agent (model sac-100, analytically pure) was purchased from Shanghai Youen Chemical Co., Ltd. (Shanghai, China). Structural adhesive (model RF-C05) was purchased from Harbin Chengfeng Adhesive Co., Ltd. (Harbin, China). Wood samples [50 mm (longitudinal) × 50 mm (tangential) × 3 mm (radial)] were used to determine resin weight percent gain (WPG) and density. The sample sizes and standards in mechanical property testing are detailed in Table 1.

### 2.2. Method

#### 2.2.1. Modification of the Wood

Firstly, 100 g of acrylic resin solids was mixed with 400 mL of deionized water, and 50 mL of triethylamine was added to dissolve them in a water bath, at 63 °C. The wood samples were dried in an oven, at 103 °C, for 24 h, and then their dry weight (W_1_) was determined. Secondly, the samples were kept under vacuum of 0.1 MPa for 40 min, following at a pressure of 0.5 MPa for 60 min, and impregnation cycle of 4 rounds. Thirdly, the resultant samples were dried via air-seasoning (moisture content 10–12%), at room temperature, and cured at 120 °C for 2 h to make resin-impregnated veneer with curing agent (W_sac_). Finally, the dried samples were weighed (W_2_), and their WPGs were calculated. For comparison, untreated samples (W_CTRL_) were prepared, and each group includes 5 parallel samples. In addition, in order to verify whether acrylic resin-impregnated laminated veneer lumber can be used as structural material for outdoor building and other industries, this experiment adopts structural adhesive to resin-impregnated veneer adhesive to obtain unmodified laminated veneer lumbers (L_CTRL_) and cured resin-laminated veneer lumbers (L_sac_).

#### 2.2.2. Performance Characterization

Acrylic resins with different solid contents of 10%, 15%, 20%, 25%, 30% were prepared. The viscosity was measured via a digital rotational viscometer (NDJ-5S Shanghai Lichen Bangxi Instrument Technology Co., Ltd., Shanghai, China). Six kinds of curing agent additions of 3%, 5%, 7%, 10%, 15% and 20% (accounting for the solid content of the resin) were set. After heat curing, alkali solution dissolution and vacuum filtration, the percentage of the remaining solid resin in their respective original weight was calculated, that is, the resin conversion rate. Poplar veneer was impregnated with selected resin under vacuum impregnation condition, and laminated veneer lumber was synthesized. The modulus of rupture (MOR), modulus of elasticity (MOE), compression strength (CS), hardness (HS) and tensile strength (TS) were measured with a universal mechanical testing machine (CMT4104 Meters). The impact bending strength (IBS) were measured with an impact toughness tester (WQ-BCSYI Wanqi). The wear test was measured with a paint film wear tester (BGD-523 BIUGED) at wear resistance rate of 50 r/min and wear ring number of 500 r. For the water absorption rate (WAR), water absorption thickness expansion rate (TSR) and water absorption width expansion rate (WSR), samples were cooked, at 63 °C, for 24 h, and the weight of the cooked plates was counted.

The morphology and structure of fracture surface were observed by SEM (JSM-6610LV, JEOL, Tokyo, Japan). The SEM was operated in vacuum mode with a working distance of 6 mm, and its detector was gaseous secondary electrons. Differential scanning calorimetry analysis was measured on sample particles (10 mg) with a compensated differential scanning calorimeter (DSC 8000 PerkinElmer). Samples were heated from 35 °C to 700 °C at a gas flow rate of 100 mL/min under nitrogen atmosphere. Each treatment was repeated 3 times, and the heat flow curve was recorded.

## 3. Results and Discussion

### 3.1. Optimization of Acrylic Resin System

#### 3.1.1. Optimization of Solid Content of Acrylic Resin Liquid

**Figure 1:**
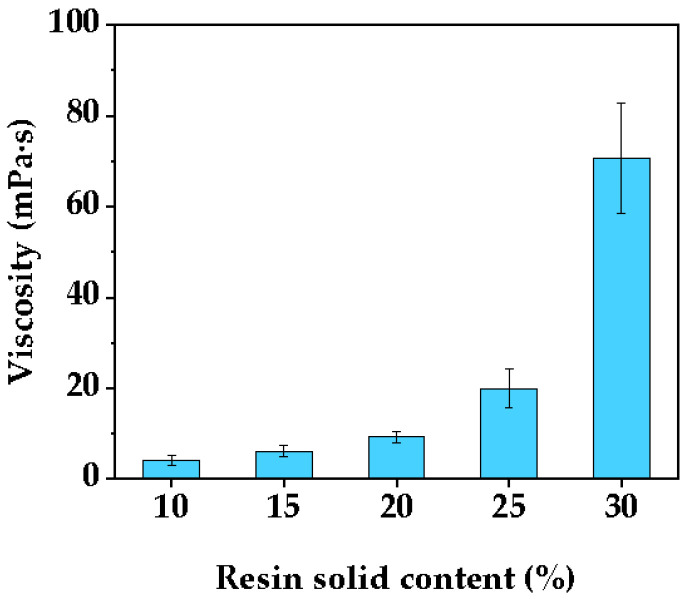
Viscosities of acrylic resin solution at different solid contents.

The values of viscosity corresponding to five different resin solid contents (10%, 15%, 20%, 25%, 30%) are presented in Figure 1. With the increase in resin solid content, the viscosity of resin increased exponentially, and the viscosity increased from 4.11 mPa·s to 9.25 mPa·s at resin solid content below 25% and increased slowly. However, when the solid content exceeded 25%, the resin viscosity increased steeply, reaching the viscosity of over 60 mPa·s at 30% solid content. Resin viscosity reflected the movement of resin molecular chains in solution. The higher the viscosity of the resin, the greater the interaction between molecular chains and the more difficult the molecular movement. In the case of fixed molecular weight of resin, as the solid content increases, the interaction between molecular chains becomes greater and the apparent viscosity increases. When the solid content exceeded a certain value, the molecular chains produce significantly increased resistance to movement due to mutual entanglement and the liquid showed a steep increase in viscosity. Generally, resin solutions with viscosities in the range of 10 to 60 mPa·s are most suitable for wood impregnation [59]. Since the higher the solid content, the higher the resin filling under the same impregnation conditions. Taking into account the suitable viscosity conditions, the solid content of the acrylic resin-impregnating solution was determined to be 25% in this study.

#### 3.1.2. Optimization of Curing Agent Addition

**Figure 2:**
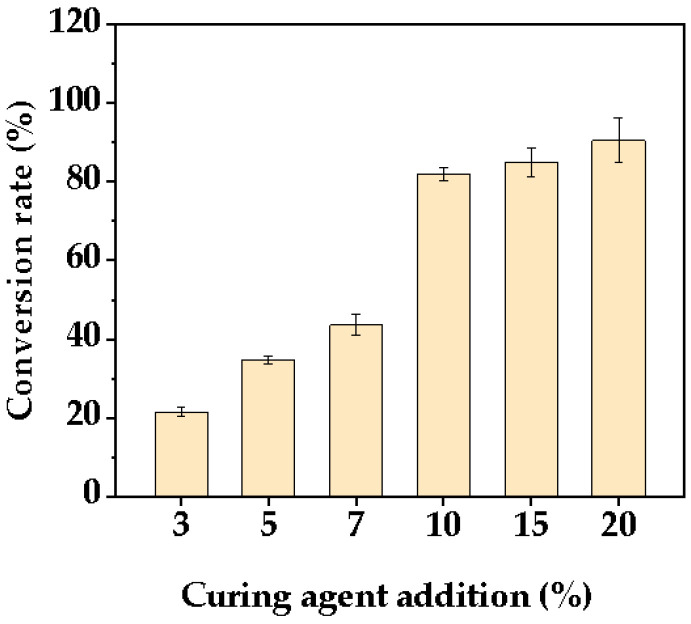
Conversion rates of resin under different curing agent additions.

Figure 2 presents the conversion values of the resin curing reaction to produce resin for different curing agent additions at 25% of the solution of acrylic resin. Overall, as the amount of hardener increased, the resin conversion rate increased. When the curing agent content was below 10%, the resin conversion rate was less than 50%. Moreover, the resin conversion rate raised steeply to 81.95% with the curing agent content reaching 10%. After that, the increase in curing agent content did not bring significant change of conversion rate. Therefore, considering the resin conversion efficiency, 10% was determined as the optimal amount of curing agent to be added.

#### 3.1.3. DSC Characterization of Acrylic Resin

**Figure 3:**
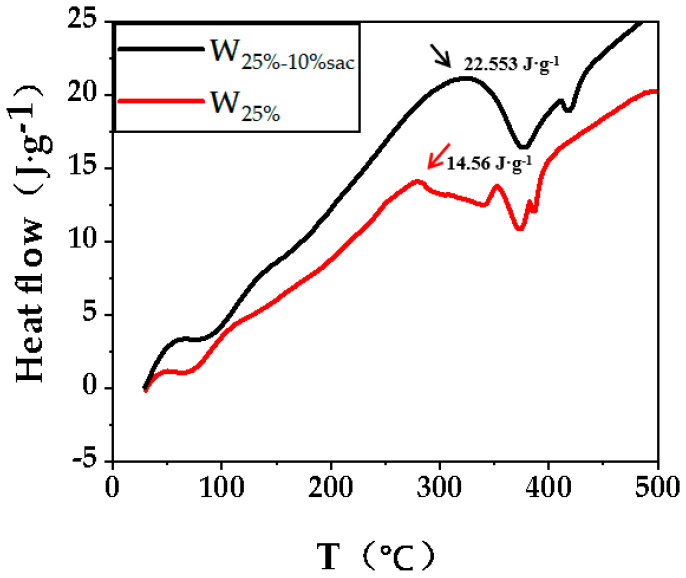
DSC curves of acrylic resin after curing.

The exothermic situation of the resin with increasing temperature with and without the addition of hardener is presented in Figure 3. Compared with the exothermic situation of the resin without curing agent, the curing window temperature range of the resin became wider (83.6~369 °C) with the addition of curing agent, and the exothermic enthalpy ΔH increased from 14.56 J·g^−1^ to 22.553 J·g^−1^, which is the result of the exothermal of the curing agent cross-linking the acrylic resin molecular chains. It can be seen that the optimized amount of curing agent will promote the cross-linking reaction of resin molecular chains, which in turn will lead to the formation of molecular cross-linked structures inside the resin and improve the macro-mechanical properties and water resistance, thus laying the foundation for the resin to fill the wood cell cavities and further improve the wood properties.

### 3.2. Performance Improvement of Poplar Veneer by Resin−Filled Cell Cavities under Optimized System

#### 3.2.1. Microstructure and Density of Resin-Filled Modified Veneer

**Figure 4:**
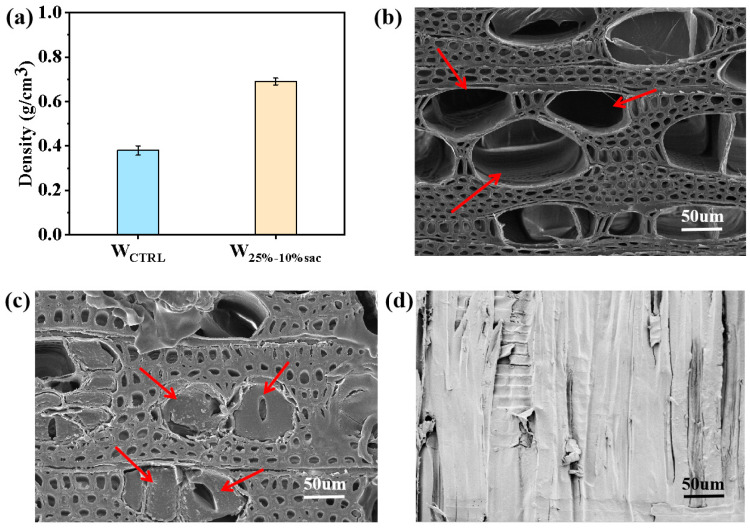
Microstructure and density of resin filled modified veneer: (**a**) Density; (**b**) SEM photo of poplar veneer cross section; (**c**) SEM photo of modified veneer cross section; (**d**) SEM photo of modified veneer longitudinal section.

The optimized resin system reacted in the cell cavity, resulting in a veneer weight gain rate of 81.36%, and at the same time, the veneer density was increased from 0.391 g/cm^3^ to 0.69 g/cm^3^ (Figure 4a), indicating that the resin solution penetrated into the cell cavity and changed from liquid to solid state through the curing cross-linking reaction, thus increasing the veneer weight. SEM images clearly showed that there was no filler inside the cell cavities of poplar veneer (Figure 4b), while in the modified veneer, a large amount of resin filled the wood cell cavities in solid state (Figure 4c,d), which is consistent with the wood density change results.

#### 3.2.2. Mechanical Properties of Resin-Filled Modified Veneer

**Figure 5:**
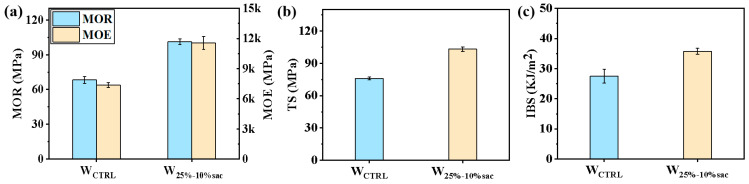
Mechanical properties of poplar veneer modified by acrylic resin filling: (**a**) MOR and MOE of veneer before and after resin treatment; (**b**) TS of veneer before and after resin treatment; (**c**) IBS of veneer before and after resin treatment.

The modulus of elasticity and the static bending strength of the resin-filled modified veneer in the optimized system were 11,563.2 MPa and 101.2 MPa which were higher than those of untreated veneer by 57.00% and 48.39% (Figure 5a). The tensile strength reached 103.2 MPa and tensile strength of 35.79% (Figure 5b), the impact toughness reached 35.78 KJ/m^2^ (Figure 5b), and the impact toughness reached 30.11% (Figure 5c). It can be seen that the optimized resin system significantly improved the static bending strength, elastic modulus, tensile strength and impact toughness of poplar veneer, which originated from the cross-linking and curing of acrylic resin in the cell cavity of wood, forming a cured resin with a certain net-like interactive structure as a reinforcing matrix, played the role of reinforcing the cell wall and then improving the stiffness and tensile resistance of the veneer. In addition, the longer molecular chains of acrylic resin are intertwined, and the chains still have a degree of flexibility. So, as a reinforcing matrix, it also presented a certain degree of toughness, which in turn improved the impact strength of the veneer.

#### 3.2.3. Dimensional Stability of Resin-Filled Modified Veneer

**Figure 6:**
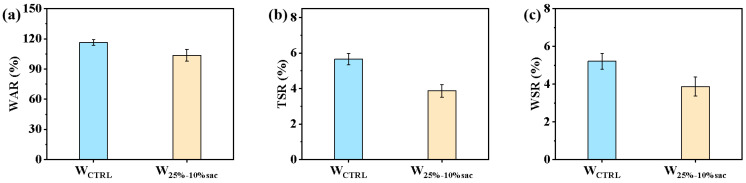
Dimensional stability of poplar veneer modified by resin filling: (**a**) Water absorption of veneer before and after resin treatment; (**b**) Water absorption thickness of veneer before and after resin treatment; (**c**) Water absorption width swelling of veneer before and after resin treatment.

The results of the water resistance test, at 63 °C, for 24 h on treated and untreated veneer showed that the water absorption of the resin-modified veneer was 103.59%, which was 10.96% less than that of the untreated veneer (116.3%). The water absorption thickness swelling was 3.87%, which was 31.50% less than that of the unmodified poplar veneer (5.65%). The water absorption width swelling was 3.87%, which was 25.72% less than that of the unmodified poplar (5.21%) (Figure 6c). This indicates that the resin filled the cell cavities and to some extent hindered the channels for water penetration into the wood, which in turn enhanced the water barrier capacity of the wood, macroscopically manifested as improved dimensional stability. The acrylic resin system curing filled the cell cavities and significantly improved the mechanical strength, wear resistance and dimensional stability of poplar veneer.

### 3.3. Performance of Laminated Composite Materials Based on Resin-Filled Modified Veneer

#### 3.3.1. Mechanical Properties of Modified Laminated Veneer Lumber

**Figure 7:**
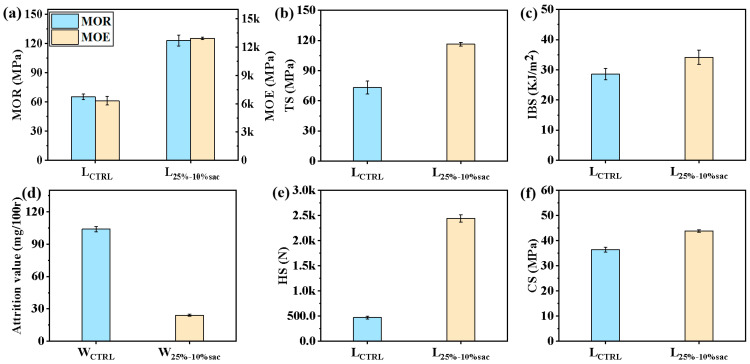
Mechanical properties of laminated composites based on resin-filled modified veneer: (**a**) MOR and MOE comparison of modified laminated veneer lumbers and untreated laminated veneer lumbers; (**b**) Modified laminated veneer lumbers and untreated laminated veneer lumbers tensile strength TS comparison; (**c**) Comparison of IBS of modified laminated veneer lumbers and untreated laminated veneer lumbers; (**d**) Comparison of attrition value of modified laminated veneer lumbers and untreated laminated veneer lumbers; (**e**) Comparison of HS of modified laminated veneer lumbers and untreated laminated veneer lumbers; (**f**) Comparison of CS of modified laminated veneer lumbers and untreated laminated veneer lumbers.

As shown in Figure 7a, the laminated veneer lumber and flexural modulus of elasticity of the laminated timber at acrylic-filled wood reached 123.13 MPa and 12,944.76 MPa, respectively, compared with the corresponding untreated veneer laminate, which increased by 88.56% and 104.79%. This is consistent with the pattern of the veneer results. The flexural strength of the modified veneer laminate is further increased compared with the corresponding static flexural strength and modulus of elasticity of the modified veneer, which is attributed to the more stable structure due to the gluing between the veneer layers, which in turn improves the structural stiffness of the overall material. Both properties exceeded the flexural strength index values (modulus of elasticity 12,500 MPa and static flexural strength 35 MPa) of structural lumber specified in the highest strength class (TC_T_40) of Chinese Standard GB 50005-2017, indicating that the flexural strength of the modified laminated veneer lumber in this study met the requirements for wood structural lumber applications.

Similarly, the tensile strength of the laminate reached 116.25 MPa with acrylic-filled wood, 58.81% higher than the corresponding untreated veneer laminate (Figure 7b). The impact toughness of the modified veneer laminate reached 34.13 KJ/m^2^, which is an increase of 19.50% (Figure 7c). Similarly to the principle of resin-reinforced veneer, resin-filled modified veneer in turn effectively improved the rigidity, toughness and tensile properties of the corresponding laminate.

In addition, the attrition value of resin-filled modified veneer was only 24.1 mg/100 r (Figure 7d), which is an improvement over untreated veneer 76.83%. This is mainly attributed to the reinforcing effect of the resin on the cell wall by cross-linking and curing in the cell cavity, contributing to the increased cohesion between the wood components. As a result, the wear resistance of the modified veneer was significantly improved.

Similarly, the hardness and compressive strength of the modified veneer laminates reached 2440.16 N and 2.47 MPa, respectively, representing an increase of 419.18% and 20.26% over the corresponding untreated laminated veneer lumber (Figure 7e,f). This also stems from the fact that the resin filling the cell cavities reinforced the wood cell walls to some extent, which in turn increased the hardness and compressive strength of the material.

#### 3.3.2. Dimensional Stability of Modified Laminated Veneer Lumber

**Figure 8:**
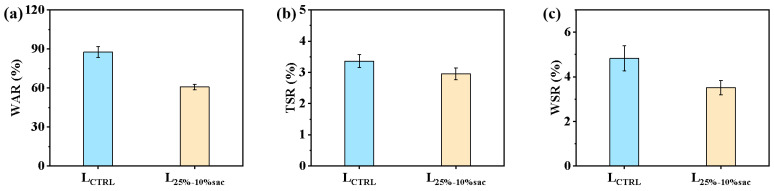
Dimensional stability of modified laminated veneer lumbers: (**a**) Water absorption of modified laminated veneer lumbers and untreated laminated veneer lumbers; (**b**) Water absorption thickness swelling of modified laminated veneer lumbers and untreated laminated veneer lumbers; (**c**) Water absorption width swelling of modified laminated veneer lumbers and untreated laminated veneer lumbers.

As shown in Figure 8a, the water absorption of the modified veneer laminate was only 60.72%, which was 44.38% better than the corresponding unmodified veneer laminate and significantly better than the water absorption of the modified veneer itself. Similarly, the absorbent thickness swelling and absorbent width swelling of the modified veneer laminate were 2.95% and 3.51%, respectively, which were 13.90% and 37.60% better than those of the corresponding untreated veneer laminate (Figure 8b,c), and also slightly better than the two properties of the corresponding modified veneer. This indicated that inter laminar gluing aided the dimensional stability of the laminated material.

On the one hand, the resin system was introduced into the cell cavity of wood, which hindered the penetration channel of water into the pores of wood, and then effectively improved the dimensional stability of laminated wood. On the other hand, the laminated glue of the veneer caused the structural adhesive to hinder the penetration of water from the surface to the internal channel of veneer, and also limited the expansion and deformation of veneer. So, the modified laminated veneer wood improved dimensional stability significantly.

This research focuses on the various properties and microscopic characterization of resin-modified laminated veneer lumber under the type and amount of the curing agent. Compared with other curing agent cross-linked acrylic resin-impregnated boards, the mechanical properties of the acrylic modified laminated veneer lumber cross-linked with pyridine curing agents were significantly improved.. Compared with polycarboxylic acids curing agent system [40], MOR in propidium curing agent system increased by 48.91%, MOE increased by 42.41%, and HS increased by 370.34%. Compared with the polycarbodiimide curing agent system [41], HS in this system increased by 294.85%. Compared with N, N-dimethylethanolamine (DMEA) and p-toluene sulfonic acid (PTSA) mixed system [44], the MOR, MOE, CS, IBS and WAR of this system increased by 3.21%, 28.98%, 154.11%, 5.31% and 47.23%, respectively. The poplar laminated veneer lumber in this study exhibits good mechanical strength and dimensional stability and is expected to be used as structural timber for building applications.

## 4. Conclusions

Although there have been some initial explorations of aldehyde-free resin-impregnated reinforced wood and achieved beneficial results, the research of acrylic resin system still needs to be further studied. To this end, we focused on the various properties and microscopic characterization of modified laminated veneer lumber under different curing agent additions, and the results are as follows:

(1) Acrylic resin-optimized system with a 25% solid content and a 10% curing agent dosage, when the resin liquid viscosity is 19.89 mPa·s, and the resin conversion rate is 81.95%.
(2) With a weight gain of 81.36% and a density of 0.69 g/cm^3^, the optimized resin system can effectively filled the poplar veneer cell lumen, which significantly improves the mechanical strength and dimensional stability of the poplar veneer.
(3) The modified laminated veneer timber’s static flexural strength and modulus of elasticity are 123.13 MPa and 12,944.76 MPa, respectively, which exceeded the flexural strength index values (modulus of elasticity 12,500 MPa and static flexural strength 35 MPa) for structural timber specified in the highest strength class (TC_T_40) of Chinese Standard GB 50005-2017. In addition, in terms of mechanical properties, compared with untreated laminated veneer, the tensile strength, impact toughness, hardness and wear resistance were 58.81%, 19.50%, 419.18%, 76.83%, respectively. In terms of dimensional stability, compared with untreated laminated veneer, the water absorption rate, water absorption thickness expansion rate and water absorption width expansion rate increased by 44.38%, 13.90% and 37.60%, respectively. It has significantly improved mechanical strength and dimensional stability, and can be used as a structural building material.

## Figures and Tables

**Table 1:** Sample sizes and standards in mechanical property testing.

Mechanical Property	Dimension l × t × r (mm)	Standard
MOR	200 × 50 × 10	GB-T 20241-2006
MOE	200 × 50 × 10	GB-T 20241-2006
CS	23 × 15 × 15	GB-T 17657-2013
HS	50 × 50 × 15	GB-T 17657-2013
IBS	300 × 20 × 20	GB-T 1938-2009
TS	408 × 25 × 25	GB-T 1938-2009
Attrition value	100 × 100 × 15	GB-T 17657-2013

## Data Availability

The data presented in this study are available on request from the corresponding author.
